# Interaction of Positively Charged Oligopeptides with Blood Plasma Proteins

**DOI:** 10.3390/ijms24032836

**Published:** 2023-02-02

**Authors:** Aleksandra Kotynia, Aleksandra Marciniak, Wojciech Kamysz, Damian Neubauer, Edward Krzyżak

**Affiliations:** 1 Department of Basic Chemical Sciences, Wroclaw Medical University, Borowska 211A, 50-556 Wroclaw, Poland; 2 Department of Inorganic Chemistry, Medical University of Gdańsk, Gen. J. Hallera 107, 80-416 Gdańsk, Poland

**Keywords:** oligopeptide, bovine serum albumin, α-acid glycoprotein, γ-globulin, quenching constant (k_q_), binding constant (K_b_), the quantity of secondary structure, the binding constants

## Abstract

In this project, we combine two areas of research, experimental characterization and molecular docking studies of the interaction of positively charged oligopeptides with crucial blood plasma proteins. The investigated peptides are rich in NH_2_ groups of amino acid side chains from Dap, Orn, Lys, and Arg residues, which are relevant in protein interaction. The peptides are 9- and 11-mer with the following sequences: (Lys-Dab-Dab-Gly-Orn-Pro-His-Lys-Arg-Lys-Dbt), (Lys-Dab-Ala-Gly-Orn-Pro-His-Lys-Arg), and (Lys-Dab-Dab-Gly-Orn-Pro-Phe(2-F)-Lys-Arg). The net charge of the compound strongly depends on the pH environment and it is an important aspect of protein binding. The studied oligopeptides exhibit therapeutic properties: anti-inflammatory activity and the capacity to diminish reactive oxygen species (ROS). Therefore, the mechanism of potential binding with blood plasma components is the next challenge. The binding interaction has been investigated under pseudo-physiological conditions with the main blood plasma proteins: albumin (BSA), α1-acid glycoprotein (AAG), and γ-globulin fraction (GGF). The biomolecular quenching constant (k_q_) and binding constant (K_b_) were obtained by fluorescence spectroscopy at various temperatures. Simultaneously, the changes in the secondary structure of proteins were monitored by circular dichroism (CD) and infrared spectroscopy (IR) by quantity analysis. Moreover, molecular docking studies were conducted to estimate the binding affinity, the binding domain, and the chemical nature of these interactions. The results show that the investigated oligopeptides could be mainly transported by albumin, and the binding domain I is the most favored cavity. The BSA and GGF are able to form stable complexes with the studied compounds as opposed to AAG. The binding reactions are spontaneous processes. The highest binding constants were determined for Lys-Dab-Dab-Gly-Orn-Pro-His-Lys-Arg-Lys-Dbt peptide, in which the values of the binding constants K_b_ to BSA and GGF were 10.1 × 10^4^ dm^3^mol^−1^ and 3.39 × 10^3^ dm^3^mol^−1^, respectively. The positively charged surface of peptides participated in salt bridge interaction with proteins; however, hydrogen bonds were also formed. The secondary structure of BSA and GGF after contact with peptides was changed. A reduction in the α-helix structure was observed with an increase in the β-sheet and β-turn and random coil structures.

## 1. Introduction

When insulin was first applied to treat diabetes, the new era of peptides as therapeutics began [1]. Nowadays, the pharmaceutical market is developing peptides or peptide-like drug fields that can be used to treat numerous diseases, e.g., diabetes, cancer, chronic pain, multiple sclerosis, HIV, and bacterial infection [2,3,4,5,6]. There are three main sources of bioactive peptides: (1) derived from natural organisms such as plants, fungi, animals, and humans (e.g., hormones, enzymes), (2) from genetic, recombinant, or synthetic chemical libraries [7,8], and (3) non-ribosomal peptides (NRPs) produced by microorganisms such as bacteria and fungi [9,10]. Peptides have many advantages: low toxicity, a relatively small molecular weight and size, high binding affinity to molecular targets, and exquisite target specificity [11,12,13]. Nevertheless, very often they possess short half-lives, are biodegradable by proteases (which limits oral administration), and have rapid renal clearance [11,12,13]. However, these inconveniences may be limited by the insertion of structural modification or by using a different method of administering the peptide-like drug. The cyclization of peptides increases biological oral ability because cyclic analogues are more protease-resistant than linear ones [14,15,16]. Moreover, backbone modification via amino acid position, type or tandem substitution, N-Alky and N-methylation amino acids, and the incorporation of D-amino acids can limit enzymatic degradation [17,18,19]. Another chemical change in peptide structure that contributes to regulating biological activity is the incorporation of unnatural residues into the sequence [20]. Unnatural amino acids are non-proteinogenic amino acids that are found in antibiotic peptides such as colistin and polymyxin B that contain five 2,4-diaminobutyric acid residues (Dab) [21]. These remaking structures allow for the modulation of pharmacokinetic properties and target specificity.

Human blood is a mixture of plasma, red blood cells, and white blood cells, and the main ingredients include water, proteins, and inorganic salts. Blood plays a crucial role in pH regulation, temperature control, and the defense against toxins or pathogens but most importantly transports nutrients, hormones, drugs, and their metabolic wastes [22]. The protein serum level is about 60–80 mg/mL, of which as much as 50–60% is albumin, and 40% is globulins [23,24]. Human albumin with a molecular weight of 66.5 kDa is a single protein chain form in a globular shape. In its structure, three homolog domains (I, II, and III) can be distinguished, which consist of two subdomains (A and B) [25,26]. There are numerous structural similarities between the human isoform of albumin (HSA) and the bovine isoform (BSA). The BSA demonstrates 76% of sequence homology with the amino acids’ order in HSA [27]. This is due to there being almost the same amino acid sequence and surface charge distribution in both structures [28]. Moreover, the globular shape and the high stability of proteins follow from the presence of seventeen intermolecular disulfide bridges in the structures. It is also related in a way to their stiffness [29] and can contribute to reducing flexibility in the binding ligands processes. For these reasons, the BSA, which is more easily available, is often used in experiments instead of HSA. The secondary structure of BSA is dominated by the α-helix structure (60–50%). Usually, the binding of small active molecules destabilizes the α-helix structure in favor of the β-sheet, β-turn or random arrangements [30,31,32,33]. In the subdomains IIA and IIIA are located two binding sites: Sudlow 1 and Sudlow 2 [34,35]. The Sudlow 1 binding site is rather an apolar pocket dedicated to such compounds as: warfarin, phenylbutazone, amantadine, azapropazone, and azidothymidine [36]. Sudlow 2 is mostly a hydrophobic site and binds such drugs as ibuprofen, digitoxin, benzodiazepine, halothane, and propofol [36]. The third binding site was identified in the IB subdomain and binds lidocaine, bilirubin, warfarin, myristic acid, naproxen, and indomethacin [35]. The α1-acid glycoprotein (AAG) is also blood plasma protein with a lower concentration than that of albumin at a level from 0.45 to 1.12 mg/mL [37]. It is an acute-phase protein whose concentration depends on various inflammatory diseases such as cancer or trauma, e.g., surgery, and the level of it may be higher, even several times larger [37]. One of the AAG physiological functions is the transportation of endogenous and exogenous compounds [38,39,40]. It is the second, after albumin, drug transport protein in the bloodstream. While HSA binds mainly to acidic substances, AAG is a carrier of basic and neutral compounds [41]. Therefore, the analysis of the interaction of new compounds with both proteins, HSA and AAG, seems to be the best way to determine its bioavailability, pharmacokinetics, or pharmacodynamics. In addition, AGP has anti-inflammatory and immunomodulatory effects. Due to the strongly changing concentration in pathological conditions, it can also be a valuable diagnostic tool [42]. The molecular weight of AAG is in the range of 41–43 kDa and consists if a polypeptide chain with two disulfide bridges that stabilize the protein structure [40,43,44]. The secondary structure of AAG is dominated by β-arrangements (41% sheets, 12% turns, and 8% bands) [39]. There is one major binding site in AAG located in the hydrophobic cavity and created by the tertiary structure. Other binding sites have low affinities and little significance [45]. The AAG binds lidocaine, propranolol, verapamil, and lipophilic molecules such as steroid hormones and also acidic drugs, i.e., phenobarbital [37,46]. The globulins also play an important role in the binding of many drugs and therapeutic agents. One of the blood fractions is globulins, which are produced by the immune system. The γ-globulins fraction (GGF) mainly consists of immunoglobulins, which are divided into five classes. The normal total concentration of IgG in the bloodstream is within 10–25 mg/mL, but in some pathological conditions, this can be much larger [47]. The γ-globulin fraction can bind a variety of metabolites, drugs, and organic compounds [48,49].

The subject of this study was oligopeptides with the following sequences: Lys-Dab-Dab-Gly-Orn-Pro-His-Lys-Arg-Lys-Dbt, Lys-Dab-Ala-Gly-Orn-Pro-His-Lys-Arg, and Lys-Dab-Dab-Gly-Orn-Pro-Phe(2-F)-Lys-Arg, which are referred to as L1, L2, and L3, respectively, in the remainder of this work. The investigated oligopeptides consist of an amino group (NH_2_) in the side chain of unique unnatural amino acids such as Dab (2,4-diaminobutyric acid) and Orn (2,5-diaminopentanoic acid) and standard amino acids such as Lys, and N-terminus and in Dbt in the C-terminal region. Additionally, the presence of the guanidino group of Arg and His moieties influence the acid/base character of the peptide and is related to further interaction with other molecules and biological fluids. The analyzed compounds can be included in the group of cationic peptides, which are one of the subgroups of antimicrobial peptides (AMPs). Thus far, several thousand compounds in this group have been identified. The positive charge of the molecules enhances the antimicrobial activity of these compounds by interacting with bacterial membranes that are negatively charged. The positive charge of AMPs is obtained by adding charge-rich amino acids to the peptide sequence, such as, for example, Lys, Arg, Orn, or Dab. Hence, the sequences of the tested peptides were designed in this way. The analyzed compounds differ in the number of amino acid residues in their sequences and the number of positively charged amino acids at the beginning and the end of the peptide chain [50]. L1, L2, and L3 were synthesized manually by the solid-phase Fmoc/tBu method. The synthesis and purification protocols were described in an earlier work [50]. Previous studies have proved that these compounds are non-toxic. They bind Cu(II) and Ni(II) ions and form thermodynamically stable complexes. Moreover, L1, L2, and L3, as well as their divalent metal complexes, reduce the level of oxygen-free radical species (ROS) and possess anti-inflammatory activity [50]. All of these findings were an inspiration for the current investigation. The studied oligopeptides in a water solution at physiological pH (7.5) contain species with a positive charge: [H_8_L1]^8+^, [H_6_L2]^5+^ and [H_5_L2]^4+^, [H_6_L3]^5+^ [50]. L. Yin and Y. Huang reported that numerous biologically active cationic peptides need to have both a charge from 3+ to 9+ and a hydrophobic component [51,52]. All investigated peptides fulfill these conditions.

The binding of a drug to a plasma protein is a major factor of the impact on pharmacokinetic (absorption, distribution, metabolism, and elimination) and pharmacodynamic (pharmacological effects) properties. There is much research on the binding of small molecules and potential new therapeutic agents by albumin but little in terms of the interaction with other components of the blood serum. Therefore, the investigated studies focus not only on the interaction with albumin (BSA) but also with other proteins in plasma such as α1-acid glycoprotein (AAG) and γ-globulin fraction (GGF). The main goal of this work is to examine the transport of possible peptide therapeutic agents by three blood plasma proteins. The knowledge of the mechanism of interaction between peptides and proteins is an insufficiently known but interesting topic nowadays. The combination of multispectroscopic experimental results is supported by theoretical molecular docking, which is an interdisciplinary approach.

## 2. Results and Discussion

### 2.1. Interactions with Albumin, α1-Acid Glycoprotein, and γ-Globulin—Spectroscopic Studies

#### 2.1.1. Fluorescence Spectroscopy

**Figure 1:**
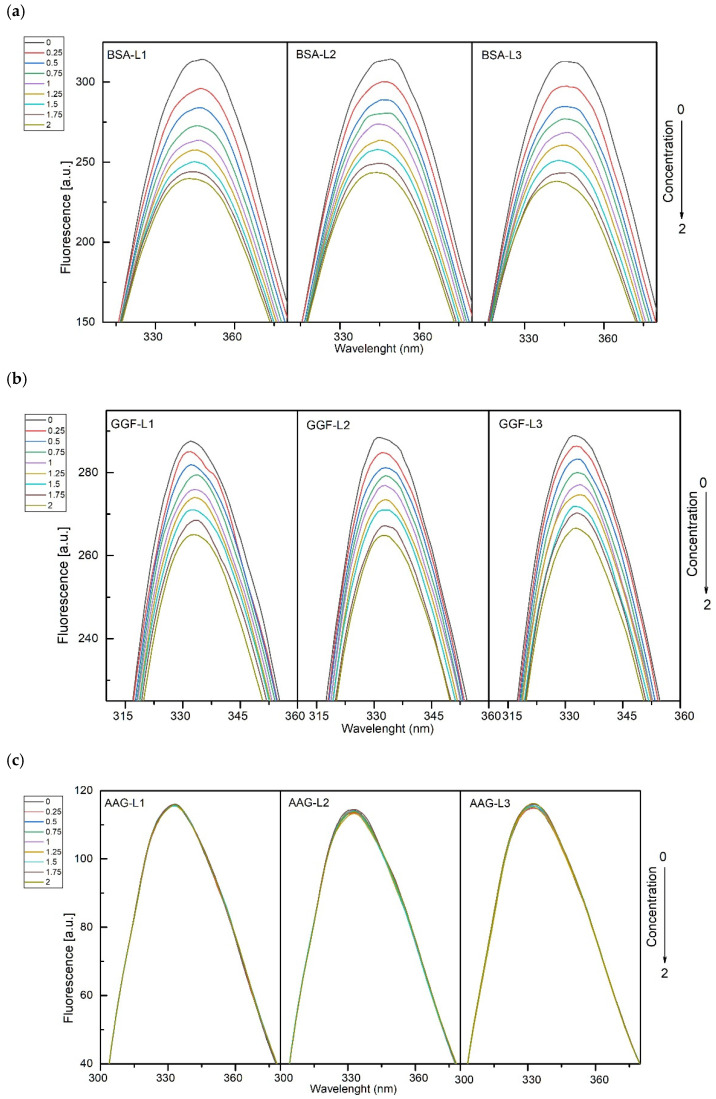
(**a**) Fluorescence plots of BSA in the presence of different concentrations L1–L3. (T-297 K, λ_ex_ = 280 nm). The concentration of BSA was 1.0 µM, the concentration of L1–L3, was: 0, 0.25, 0.50, 0.75, 1.00, 1.25, 1.50, 1.75, 2.00 µM. (**b**) Fluorescence plots of GGF in the presence of different concentrations L1–L3. (T-297 K, λ_ex_ = 280 nm). The concentration of GGF was 1.0 µM, the concentration of L1–L3, was: 0, 0.25, 0.50, 0.75, 1.00, 1.25, 1.50, 1.75, 2.00 µM. (**c**) Fluorescence plots of AAG in the presence of different concentrations L1–L3. (T-297 K, λ_ex_ = 280 nm). The concentration of AAG was 1.0 µM, the concentration of L1–L3, was: 0, 0.25, 0.50, 0.75, 1.00, 1.25, 1.50, 1.75, 2.00 µM.

Fluorescence spectroscopy was carried out to characterize the mechanism of the interaction between three plasma proteins, BSA, AAG, and GGF, with the studied peptides. The fluorescence spectra of protein were collected during titration of L1–L3. The excitation wavelength was set at λ = 280 nm (both Trp and Tyr residues were excited). The fluorescence plots are presented in Figure 1a–c. For L1–3/BSA and L1–3/GGF systems, the results indicate interactions between proteins and peptides. With increasing L1–L3 concentrations, the fluorescence intensity decreases. A blue shift in the maximum of the emission peak is also observed. This suggests that the microenvironment around the chromophore of proteins changes and the amino acid residues are located in a more hydrophobic environment [53]. For the L1–3/AAG systems, as shown in Figure 1c, fluorescence intensity upon titration of L1–L3 hardly changes. This suggests a very weak interaction as a result of the collisional encounters.

The fluorescence quenching and shift of λ_max_ for interactions with serum albumin and γ-globulin can suggest the static mechanism and formation of a stable complex. To confirm this, the fluorescence data were further analyzed by the Stern–Volmer Equation (1) after correction due to the infer filter effect (2):

(1) $$\frac{F_{0}}{F}=1+k_{q}\tau \left[Q\right]=1+K_{\mathrm{SV}}\;$$

(2) $$F_{\mathrm{corr}}=F_{\mathrm{obs}}10^{\frac{\left(A_{\mathrm{ex}}+A_{\mathrm{em}}\right)}{2}}$$

where F_0_ and F are the fluorescence intensities (after correction) of the protein and protein in the presence of L1–3, respectively, k_q_ is the quenching rate constant, τ_0_ the average lifetime, [Q] is the L1–3 concentration, K_sv_ is the Stern–Volmer constant, and F_corr,_ and F_obs_ are the corrected and observed fluorescence intensities, respectively. A_ex_ and A_em_ are the absorbance values at excitation and emission wavelengths, respectively. A linear regression fit was used to calculate the value of the Stern–Volmer constant (K_sv_). Then, assuming the average lifetime of the fluorophore in the excited state for a biomolecule as 10^−8^ s [54], the quenching rate constant (k_q_) was computed. For dynamic quenching, the maximum scatters collision quenching rate constant for different quenchers with the biopolymers was reported to be 2 × 10^10^ dm^3^mol^−1^s^−1^ [55,56]. As presented in Table 1, k_q_ for interactions with BSA is 1.55 × 10^13^ dm^3^mol^−1^s^−1^ for the L1/BSA system, 1.41 × 10^13^ dm^3^mol^−1^s^−1^ for L2/BSA, and 1.53 × 10^13^ dm^3^mol^−1^s^−1^ for L3/BSA. For interactions with γ-globulin fraction, k_q_ is smaller: 4.29 × 10^12^ dm^3^mol^−1^s^−1^ for L1, 3.99 × 10^12^ dm^3^mol^−1^s^−1^ for L2, and 3.87 × 10^12^ dm^3^mol^−1^s^−1^ for L3 (Table 2). However, the obtained values are much greater than the maximum scatters collision. It indicates a static mechanism and the formation of a stable complex with the protein. Collisional quenching occurs when the excited-state fluorophore is deactivated by contact with some other molecule in solution (by collisions). The molecules do not form a stable complex. This is a dynamic mechanism of quenching. The quenching constant characterizes the quenching mechanism. The maximum value for collisional quenching is 2 × 10^10^ dm^3^mol^−1^s^−1^. If the quenching constant is higher, it suggests a static mechanism of quenching and complex formation. For further confirmation, measurements were made at, in addition to 297 K, temperatures 303 and 308 K. Experimental data points were fitted and analyzed using Equation (1). The results are listed in Table 1 and Table 2. The calculated Stern–Volmer constant and quenching rate constant decrease with increasing temperature. It clearly indicates that peptides L1, L2, and L3 quench the fluorescence of BSA and GGF through a static quenching mechanism rather than a dynamic one.

**Figure 2:**
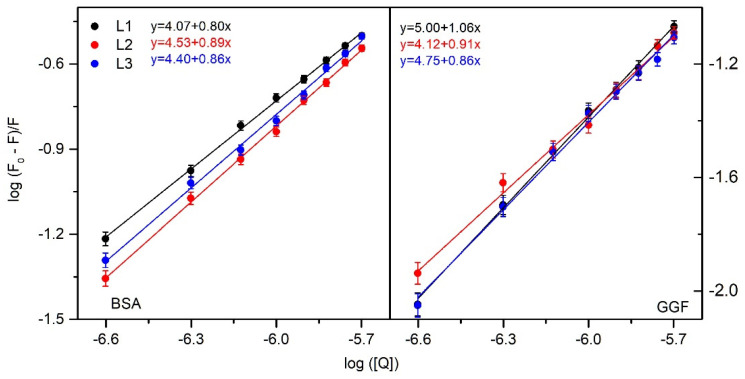
Double logarithm regression plots for quenching of BSA (**left**) and GGF (**right**) by peptide L1–3.

Both for the complexes with BSA and with GGF, the binding constant (K_b_) and the binding stoichiometry (n) were calculated. A double log regression curve was used to fit the experimental data, according to Equation (3):

(3) $$\log\frac{F_{0}-F}{F}={\mathrm{logK}}_{b}+\mathrm{nlog}\left[Q\right]$$

where F_0_ and F are the fluorescence, after correction due to the infer filter effect, in the absence and presence of peptide L1–3, respectively, and [Q] is the peptide concentration. The values of K_b_ and n were calculated from the slope and the intercept of the log [(F_0_ − F)/F] versus log [Q] plot (Figure 2). The obtained values are collected in Table 1 for the L1–3/BSA complexes and Table 2 for L1–3/GGF. For the interactions with serum albumin, K_b_ is 1.17 × 10^4^ for L1, 3.39 × 10^4^ for L2, and 2.52 × 10^4^ for L3. The binding stoichiometry (n) is 0.8–0.9, suggesting one-to-one interaction. For the interactions with γ-globulin, K_b_ values are similar to BSA complexes: 1.01 × 10^5^ for L1, 1.32 × 10^4^ for L2, and 5.63 × 10^4^ for L3, with n close to 1. The structural modification does not affect the binding constant much. The results indicate that the interaction with BSA and GGF is rather moderate, allowing both transport and release from the complex into the bloodstream.

The forces involved in the interaction can be identified by measurement at different temperatures and then by thermodynamic analysis. The signs of the thermodynamic parameters identify the type of interactions [57]. The enthalpy change (ΔH°), the entropic change (ΔS°), and the free energy change (ΔG°) were calculated from Equations (4) and (5):

(4) $${\mathrm{logK}}_{b}=-\frac{\Delta H^{{}^{\circ}}}{\mathrm{RT}}+\frac{\Delta S^{{}^{\circ}}}{R}$$

(5) $$\Delta G^{{}^{\circ}}=\Delta H^{{}^{\circ}}-T\Delta S^{{}^{\circ}}=-{\mathrm{RTlnK}}_{b}$$

where K_b_ is the binding constant and R is the universal gas constant. The calculated parameters are presented in Table 1 and Table 2. To determine the thermodynamic parameters, the van’t Hoff plot was first conducted, i.e., the temperature dependence of the binding constant, according to Equation (4). The K_b_ values at the three temperatures were fitted by the linear regression method. The values of ΔH and ΔS were calculated from the slope and intercept at the fitted line. Next, the free energy change (ΔG°) was obtained from Equation (5). The non-covalent interaction between the protein and the ligand stabilized the complex structure. The confirmation of the binding mode was performed by the analysis of the thermodynamic parameters. Generally, the positive or negative values of enthalpic and entropic change (ΔH°, ΔS°) indicate the binding mode to be a hydrophobic interaction (ΔH° > 0, ΔS° > 0), only an electrostatic interaction (ΔH°~0, ΔS° > 0) and hydrogen bonding, or/and van der Waals force (ΔH° < 0, ΔS° < 0) [57]. The results show that the interaction between the studied peptides and BSA and GGF is spontaneous due to the negative ΔG° values. Furthermore, both the ΔH° and ΔS° negative values indicate that the main interaction in the binding process is a van der Waals forces and/or hydrogen bonding interaction. The obtained results of the thermodynamic constants for the BSA complexes are comparable with the literature data obtained for anti-inflammatory compounds of pyridazone derivatives. The series of N-substituted-1,2,4-triazole-based derivatives of pyrrolo [3,4-d]pyridazinone exhibit comparable binding constants of K_b_ direct to BSA such as the studied ligands, and are able to fit to one binding site, named site II. The thermodynamic parameters for Gibbs energy (ΔG°) for pyridazinone analogues are within the range from −26.2 to −28.9 kJmol^−1^ with simultaneously negative values of ΔH° and ΔS° [58]. The tyrosinase inhibitors (TKIs), gefitinib, lapatinib, and sunitinib, which displayed antitumor activity, interact with BSA with one-to-one stoichiometry where the K_b_ constants are 8.32 × 10^4^ dm^3^mol^−1^, 2.24 × 10^5^ dm^3^mol^−1^, and 1.32 × 10^5^ dm^3^mol^−1^ at 298 K, respectively. The formation of BSA complexes is a spontaneous process and all thermodynamic parameters move toward negative values [59].

To check if L1–L3 bind to BSA at the two Sudlow drug sites (site 1 and 2 [34,60]), displacement studies were conducted by using phenylbutazone (PHB) and ibuprofen (IBP) as site probes. Site 1 shows the binding affinity towards PHB, and site 2 is known to bind IBP [60]. The fluorescence emission spectra of BSA and site markers during the titration of L1–L3 were recorded. The experimental data were analyzed using Equation (3). Binding constants in the presence of site markers were calculated and compared with K_b_ without PHB or IBP. The results are listed in Table 3. The results show that K_b_ in the presence of PHB or IBP slightly decrease compared to BSA alone. It suggests that site 1 and site 2 are actually not preferred, and ligands L1–3 anchor elsewhere, e.g., into the pocket in domain I (site 3).

#### 2.1.2. CD Spectroscopy

**Figure 3:**
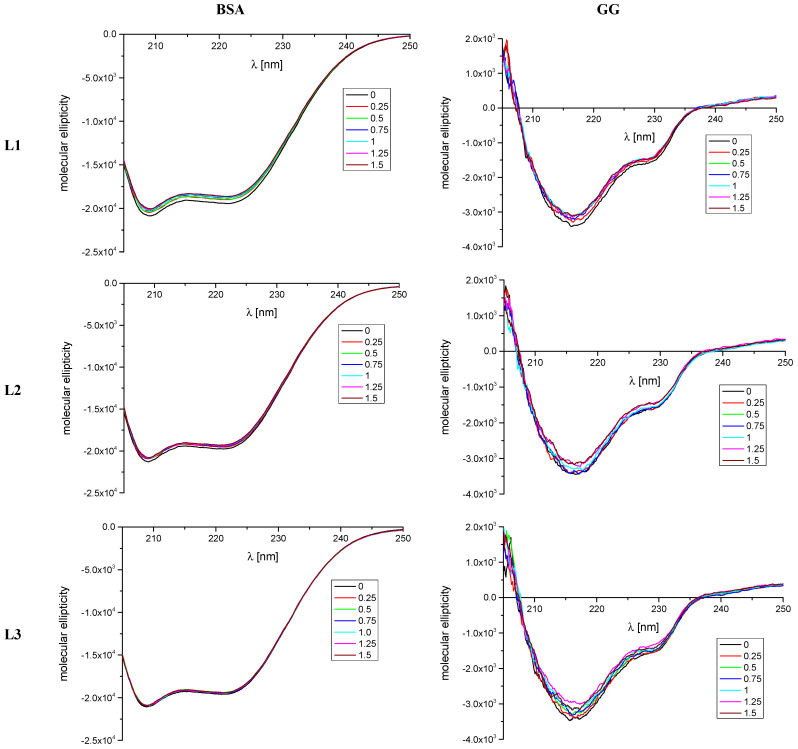
Circular dichroism spectra of BSA and GGF in the absence and presence of peptides L1, L2, and L3. Molecular ellipticity was calculated using mean residue molar concentrations.

According to fluorescence spectroscopy experiments, the studied oligopeptides did not have significant interaction with AAG. Considering these results, it was decided to limit further research to interactions with BSA and GGF. Circular dichroism spectroscopy, similar to fluorescence and FTIR spectroscopies, is a useful method to determine the changes in the secondary structure of proteins in case of the presence of compounds that can interact with protein molecules [61,62]. Therefore, in this study, the CD spectra for BSA protein and GGF fraction, in the absence and presence of analyzed peptides L1, L2, and L3, were measured (Figure 3). We wanted to check the changes in the course of the spectrum after adding every portion of the analyzed peptides, from 1:0 to 1:1.5 protein to L1–L3 molar ratios. The obtained results were analyzed by the CD Multivariate SSE program, and are summarized in Table 4 and Table 5.

The obtained CD spectra are characteristic of the analyzed proteins. For BSA, two negative α-helix bands near 209 and 220 nm are present [63] (Figure 3). The changes observed in the spectrum during the addition of successive portions of the test compounds are the largest for L1 and equal to 2.5% for α-helix content (Table 4). There is also a minor reduction in the percentage of α-helix in the case of L2 and L3 amounting to 1.2% and 0.4%, respectively (Table 4). However, the course of the spectrum and the position of the bands do not change. Only the intensity of the bands is slightly reduced. Therefore, it can be concluded that the analyzed peptides interact with the BSA molecule; however, this interaction does not significantly destabilize the protein structure.

Two negative bands near 215 and 230 nm are observed in the GGF spectra (Figure 3). After the addition of each aliquot of the analyzed peptides, the intensity of the bands observed in the spectra is reduced. However, similar to BSA, the course of the spectrum and the position of the bands do not change. The β-sheet is the predominant secondary structure for this protein (Table 5). However, the results obtained show that with the increasing concentration of the analyzed peptides there are slight changes in the percentage for this form. In the case of the α-helix content, the changes that occur do not exceed 1.2% and are the largest for L3. It can therefore, again, be concluded that the analyzed peptides interact with the GGF fraction; however, this interaction does not significantly destabilize the protein structure.

#### 2.1.3. ATR-FTIR Spectroscopy

**Figure 4:**
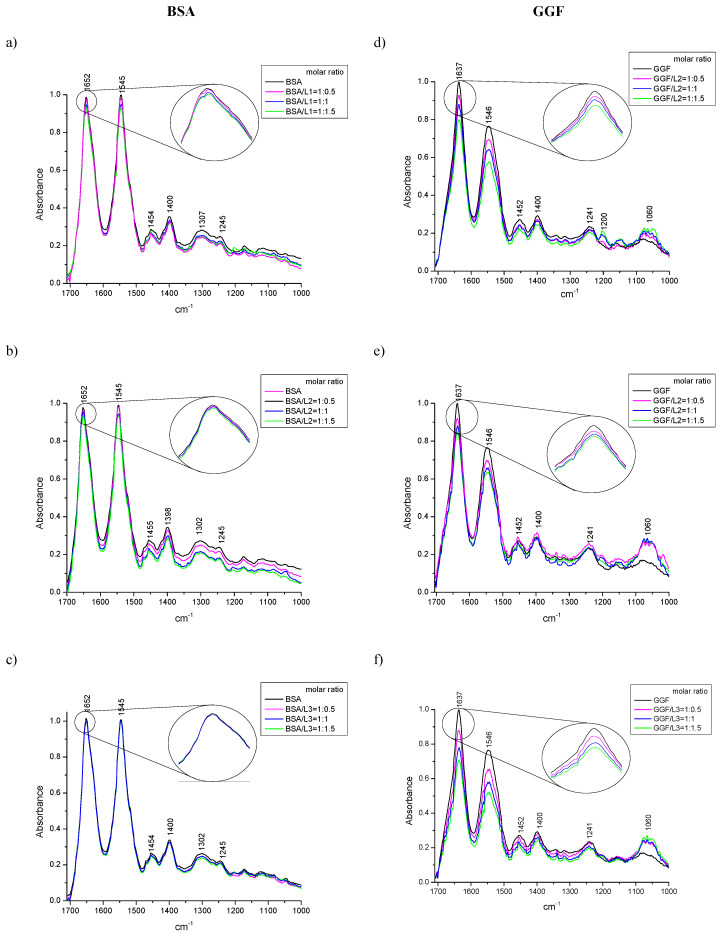
The ATR-FTIR spectra of free BSA, GGF, and with added oligopeptides where the molar ratio protein/peptide was 1:0.5, 1:1, 1:1.5, the concentration of protein was 6.5 ×10^−4^ M, (**a**) BSA/L1, (**b**) BSA/L2, (**c**) BSA/L3, (**d**) GGF/L1, (**e**) GGF/L2, (**f**) GGF/L3.

The perturbation in protein structure conformation can be monitored by CD spectroscopy as well as the ATR-FTIR method. These secondary structure changes are confirmation of the interaction between the studied compound and the protein [64,65,66,67]. The range of the wavenumber from 1700 to 1000 cm^−1^ is the most varied region and with the richest information on protein spectra. The most characteristic bands are Amide I and Amide II, which originate from the C=O stretching vibration and C–N– stretching coupled with N–H bending, respectively [68,69]. Amide I was detected for BSA at 1652 cm^−1^ and GGF at 1637 cm^−1^ (Figure 4). The differences in the position of these bands are connected with the variety in the secondary structure conformation, while Amide II consistently occurred at 1545 and 1546 cm^−1^ on the BSA and GGF spectra, respectively (Figure 4). The much less intense peak of Amide III usually accrued by C–N– stretching and bending vibration was only noticed in the BSA spectrum at 1307 cm^−1^ (Figure 4). The peaks detected at 1454 cm^−1^ (BSA) and 1452 cm^−1^ (GGF) appeared by CH_3_ group symmetric and asymmetric bending vibration (δ_s_, δ_as_). The absorption signal 1400 cm^−1^ may be assigned to stretching vibration (ν) caused by the carboxylic group (COO^−^). The studied peptides interact with BSA and GGF, which is manifested by a reduction in the intensity of the absorption peaks (Figure 4). With the increase in the ligand concentration in the protein solution, the intensity of the absorption peaks decreased (Figure 4). The Amide I and II in the BSA spectrum decreased above all after binding with L1 and slightly in the samples from L2 and L3. The major changes in the intensity absorption of Amide I and II in GGF were observed for L3, next for L1, and then for L2. Moreover, after the complexation of the investigated peptides with GGF, an increase in the band 1060 cm^−1^ was responsible for stretching vibration C–O absorption, which was manifested the most by L2 and L3 compounds.

**Figure 5:**
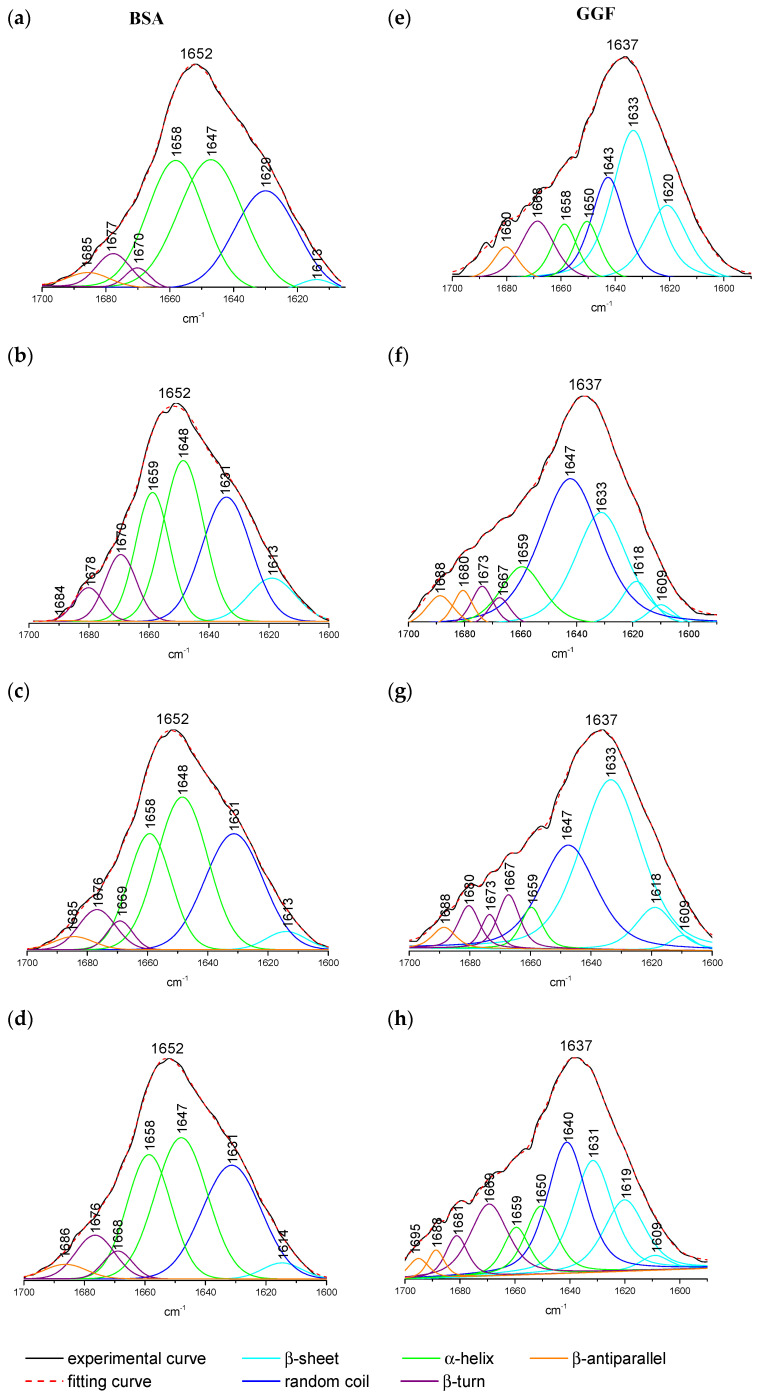
The fitting of Amide I peak of free (**a**) BSA, (**e**) GGF, and with added oligopeptides with equimolar ratio: (**b**) BSA/L1, (**c**) BSA/L2, (**d**) BSA/L3, (**f**) GGF/L1, (**g**) GGF/L2, (**h**) GGF/L3.

The shape and intensity variation in the Amide I band is the confirmation of peptide L1, L2, and L3 interaction with the protein’s backbone. The hydrogen bonds in the protein structure are violated after the active molecules’ binding and influence the frequency of the C=O vibrations’ absorption. The frequency of stretching vibrations can be reduced after changes in hydrogen bonding in the protein, whereas the frequency of bending vibrations can be increased after ligand to protein complexation [70]. The Byler and Susi procedure was used to analyse the secondary structure protein conformation [68]. The quantity analysis of the secondary structure in the protein is mostly conducted based on Amide I deconvolution, and it is the most frequently analyzed vibration band, which is very vulnerable to the detection of changes in the structure [71]. The fragment of each normalized spectrum with the Amide I peak was extracted and a second derivate was made. The self-deconvolution and curve fitting by the Gaussian/Lorenzian function allowed determination of the intensity and total area under the peaks, which corresponds with the contribution of the type of structure. The position of each band component may be assigned to a corresponding shape of the secondary structure: the α-helix (1660–1647 cm^−1^), β-sheet (1640–1610 cm^−1^), β-turn (1660–1680 cm^−1^), β-antiparallel (1681–1695 cm^−1^), and random coil (1650–1630 cm^−1^) structures [68,72,73,74]. The data results are collected in Table 6 and presented in Figure 5.

The self-deconvolution of Amide I bonds for free plasma proteins and peptide complexes is presented in Figure 5, and the percentage of changes in the secondary structure is collected in Table 6. The conformation of BSA is mostly dominated by α-helix (63.71%), and additionally contains an approximately random coil, 25.62%, a few percentages of β-turn (6.65%) and β-sheet (1.64%), and about 2.38% of β-antiparallel shape. In contrast, GGF is structurally a more diverse protein. The β-sheet structure predominates, which is about 47.7% in protein solution, then a 14.17% share of random coil. The α-helix is 14.17%, similar to β-turn 14.55%, and β-antiparallel makes up about 5.73%. The complexation of L1 to albumin showed different fluctuations in structure perturbations than for L2 and L3, which exhibited congruous tendency. The albumin structure during peptides’ interaction caused the reduction in the α-helix. For L2 and L3, it was only about 9%, but for L1 it was more than 14%. This reduction was in favor, and the growth of 1.36–3.50% of the β shape (β-sheet and β-turn), in the case of L1, showed greater differences of 7.05% and 7.34%, respectively. The β-antiparallel framework after binding L2 and L3 stayed almost at a constant level, and in complex BSA/L1, it decreased by about 2%. An increase in the share of the random structure was observed by about 4% after the addition of L2 and L3 ligands, and only by 2% after mixing the protein solution with L1. The interaction of ligand L1 with GGF plasma fraction induced destabilization of the α-helix and all β structures (shared loss of ~4% each of construction) in favor of a random coil structure (rise of ~22.9%). A very similar tendency was detected after binding the L2 peptide to GGF. The structural rearrangement from the α-helix, β-sheet (fall ~4%), β-turn (fall ~6.2%), and β-antiparallel (fall 1.4%) led to an increasing percentage in the random coil pattern (rise ~16%). A different behavior was detected for the GGF/L3 layout. The binding process reduced the percentage of the α-helix (fall ~8%) as well as β-sheet (fall ~4%), and β-antiparallel (fall 0.5%), for the benefit of the growth of form β-turn (rise ~4%) and random coil (rise ~6%).

The general conclusion of the changes in the conformational structure of blood proteins after the addition of oligopeptides is that compound L1 caused the greatest turmoil in secondary protein structure after contact with albumin (BSA). It is demonstrated by the lower intensity of Amide I and with the shape changes in this band during titration by the ligand. The changes touch on 16% of the differences in the secondary structure and contributed the most to α-helix destabilization, whereas the formation of complexes with GGF and compound L3 caused the biggest reduction in the absorbance band intensity of Amide I. Despite this, most variations in the percentage γ-globulin (GGF) secondary structure were detected in the sample after L1 addition (22.98% differences).

### 2.2. Interactions with Albumin and γ-Globulin—Molecular Docking Studies

As shown by spectroscopic studies, the interactions of the L1–L3 compounds with albumin and γ-globulin have a static mechanism. To analyze how L1–L3 bind to these proteins, molecular docking studies were performed. The crystal structures of serum albumin, PDB entry 3V03 [75], and γ-globulin, PDB entry 1AJ7 [76], were used for modeling.

**Figure 6:**
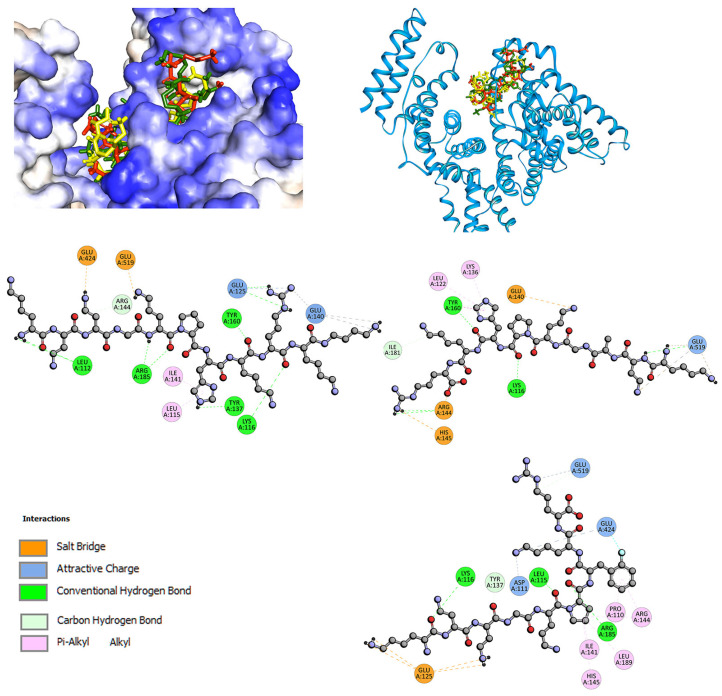
The docked pose of L1 (green), L2 (yellow), L3 (red) into site 3 of BSA and 2D interaction plot (L1, **left**; L2, **right**; L3, **right bottom**).

For interactions with serum albumin, fluorescence quenching experiments indicated that in the presence of two site markers, phenylbutazone (PHB) and ibuprofen (IBP), the binding constant for BSA/L1–L3 systems slightly decreases (Table 7). This result suggests low affinity for the interaction inside the pocket, where PHB and IBP bind. Therefore, docking studies were carried out on three subdomains [36,75,77,78]. The results are collected in Table 7. Negative binding energies were obtained at each docking, suggesting that L1–L3 can bind to multiple pocket sites: two Sudlow sites (sites 1 and 2 [34,60]) and site 3 in domain I. However, the highest affinity was observed for the interaction in the pocket of domain I: −5.5 kcal/mol for L1, −6.1 kcal/mol for L2, and −6.2 kcal/mol for L3. This is a long hydrophobic channel where long chain fatty acids bind [77,79], or several clinical drugs [80]. However, the difference in binding affinity between sites 3 and 2 is not great: only 0.2 kcal/mol for L1 and L2, and 0.4 kcal/mol for L3. When the pocket in domain I is blocked by fatty acids, the studied compounds can interact with site 2. Site 2 also binds some synthetic cationic antimicrobial peptides (CAP) [81]. Site 1 in domain II is the least preferred (Table 7). Figure 6 presents the interactions and position of compounds L1–L3 in site 3 in domain I. For all ligands, cationic regions are involved in interactions with serum albumin. Several hydrogen bonds are observed. Leu112 interacts with N-terminal amine N-For L1, Arg185 with a peptide bond, Tyr160, and Lys116 with the oxygen of the carbonyl group, Tyr137 with NH of the imidazole ring of the histidine side chain. Salt bridge contacts are observed between Glu residues and non-proteinogenic amino acids, Dab and Orn, and similarly with the guanidine group of the side chain of Arg and with 1,4-diaminobutane (Dbt) on C-terminal. Additionally, the L1-BSA complex is stabilized by π-alkyl interaction with the imidazole ring of the histidine side chain. L2 and L3 in a similar manner interact with albumin. Hydrogen bonds, salt bridge, π-alkyl, or hydrophobic interactions with alkyl alkyl group contacts are observed. Hydrogen bonds are formed with the oxygen of the carbonyl group of the peptide chain. The cationic area of Dab, Orn, and Lys are involved in salt bridge contacts. Proline and phenylalanine rings interact by π-alkyl or alkyl contacts. Further details are shown in Figure 6.

**Figure 7:**
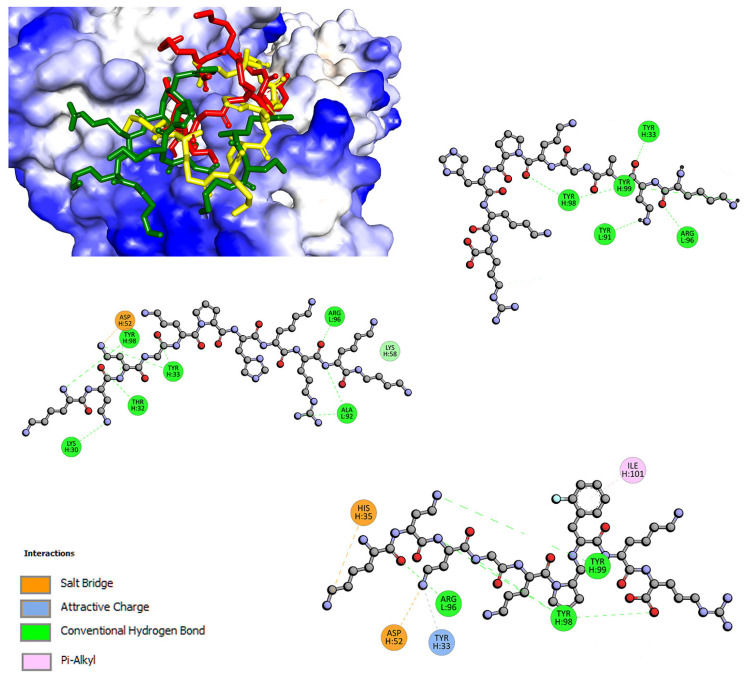
The docked pose of L1 (green), L2 (yellow), L3 (red), and 2D interaction plot (L1, **left**; L2, **right top**; L3, **right bottom**) with γ-globulin fraction.

Fluorescence studies indicated that all tested compounds form stable complexes with γ-globulin. Molecular docking shows that the binding affinity is negative, −5.5 kcal/mol for L1, −6.1 kcal/mol for L2, −5.4 kcal/mol for L3 (Table 7). The binding site of γ-globulin is a shallow cavity where ligands can interact by hydrogen bond, Van der Waals force, electrostatic interaction, and hydrophobic interaction [49]. As shown in Figure 7, part of the compound is anchored inside an active pocket. For ligand L1, with C-terminal blocked by Dbt, Arg-Lys-Dbt moiety docks in the pocket. Three hydrogen bonds with Ala92 and Arg96 are formed. The complex is also stabilized by additional H-bonds and salt bridge contacts, via Lys-Dab-Dab moiety (Figure 7). L1 and L2, with the negative partial charge at C-terminal [50], and N-terminal moiety (Lys-Dab, Lys-Dab-Dab respectively) dock into the cavity. Hydrogen bonds with Tyr33, 91, 98, 99, and Arg96 are formed (Figure 7). For L3, with fluorophenylalanine instead of histidine, π-alkyl and salt bridge are observed.

We also wanted to mention that our molecular docking studies have some limitations of docking accuracy. We used the AutoDockVina software, mainly dedicated to the interaction of small molecules with peptides. It would be best to use software for peptide–protein or protein–protein interactions, with a global docking method and flexible peptide and protein. For example, CABS-dock, pepATTRACT, PIPERFlexPepDock, Cluspro, Haddock, etc. However, all the software we wanted to use for docking have a limitation, which was a problem for us: only peptides with natural amino acids could be used, not modified. There is no support for 2,4-diaminobutyric acid (Dab) or 2,5-diaminopentanoic acid (Orn). Thus, we decided to use a tool designed for docking small molecules. We chose AutoDock Vina based on the publications where it was used to study short peptide–protein interactions [82,83,84,85].

## 3. Materials and Methods

### 3.1. Fluorescence Spectroscopy

All the fluorescence measurements were performed on a Cary Eclipse 500 spectrophotometer (Agilent, Santa Clara, CA, USA). The interaction between peptides L1–3 and bovine serum albumin (BSA) (a lyophilized powder purity ≥ 98, Sigma Aldrich, St. Louis, MO, USA), α1-acid glycoprotein (AAG) (a lyophilized powder purity ≥ 99, Sigma Aldrich, St. Louis, MO, USA), and γ-globulin fraction (GGF) (a lyophilized powder purity ≥ 98, Sigma Aldrich, St. Louis, MO, USA) was studied in phosphate buffer as a solvent and a concentration of proteins 1.0 × 10^−6^ moldm^−3^. A solution of proteins was titrated by successive additions of 1.0 × 10^−3^ moldm^−3^ solution of studied compounds, to give a final concentration of 0.25 × 10^−6^–2.0 × 10^−6^ moldm^−3^. All experiments were measured at three temperatures: 297, 303, and 308 K. Fluorescence quenching spectra were obtained at excitation and an emission wavelength of 280 nm and 300–500 nm, respectively. The molar ratio of peptides to protein was 0.25–2.0 with 0.25 steps. Binding displacement studies were carried out in the presence of the two site markers, phenylbutazone (PHB) and ibuprofen (IBP), as sites I and II markers, respectively. Concentrations of BSA and site markers were set at 5.0 × 10^−6^ and 10.0 × 10^−6^ moldm^−3^, respectively.

### 3.2. CD Spectroscopy

Circular dichroism (CD) spectroscopy was performed using the Jasco J-1500 magnetic circular dichroism spectrometer (JASCO International CO., Tokyo, Japan). All measurements for the protein solutions in the absence and presence of the analyzed peptides L1, L2, and L3 were made at room temperature under simulated physiological conditions in pH 7.4, in phosphate buffer as a solvent. The phosphate buffer not only controls the stable pH but also imitates the biological environments. However, its use leads to some limitations for CD measurements due to containing NaCl and KCl. The spectra were baseline corrected (phosphate buffer was used as a baseline) and were measured in the range of 205–250 nm at a scan rate speed of 50 nm/min, with a response time of 1 s, and a 10 mm or 5 mm path length for BSA and GGF, respectively. The concentrations of proteins and analyzed peptides were 1 × 10^−6^ mol/dm^3^ and 1 × 10^−3^ mol/dm^3^, respectively. Experiments were performed with protein to ligand molar ratios: 1:0, 1:0.25, 1:0.5, 1:0.75, 1:1, and 1:1.5. In total, 3 cm^3^ of a solution of each protein was titrated by successive additions of analyzed peptides. The results were analyzed by CD Multivariate Calibration Creation and CD Multivariate SSE programs (JASCO International CO., Tokyo, Japan), with the conversion of protein concentrations for mean residue molar concentrations.

### 3.3. ATR-FTIR Spectroscopy

The infrared spectra were measured on Nicolet iS50 FTIR (Thermo Scientific, Waltham, MA, USA) spectrometer equipped with Attenuated Total Reflectance (ATR) accessory. The deuterated triglicyne sulphate (DTGS) detector and KBr beam splitter were applied. The spectral data were recorded within the range of 4000 to 600 cm^−1^ with a resolution of 4 cm^−1^ and 100 scans were averaged for each spectrum. The measurements were accomplished at room temperature. The concentration of blood plasma proteins BSA (Sigma Aldrich, USA), and GGF (Sigma Aldrich, USA) were 6.5 × 10^−4^ moldm^−3^. The solutions of the studied peptides were 0.01 moldm^−3^. The protein BSA or GGF was mixed with an appropriate amount of compounds stock solution to achieve a 1:1 molar ratio and the spectra were registered. The ATR-FTIR spectra analysis was proceeded by Omnic 9.3.30 (Thermo Fisher Scientific Inc., Waltham, MA, USA) software and OriginPro (OriginLab Corporation, Northampton, MA, USA). The quantitative analysis of secondary structure conformation was evaluated by Byler and Susi procedure [68].

### 3.4. Molecular Docking

The crystal structures of serum albumin (3V03) and γ-globulin (1AJ7) were taken from Protein Data Bank (http://www.rcsb.org, accessed on 5 May 2022). All the ligands and water molecules were removed and then polar hydrogen atoms and Kollman charges were added using AutoDock Tools 1.5.6 [86]. The structures of the L1–L3 were optimized using PM6 methods. Calculations were performed using the Gaussian 2016 A.03 software package [87]. The molecular docking study was carried out using AutoDockVina 1.1.2 [88]. For peptides, rotatable bonds were assigned. Local docking procedure and Monte-Carlo-based sampling of peptide conformations inside binding pocket were used. The studied compounds exhibit more than 32 rotatable bonds, which is the limit in AutoDock Vina; therefore, we modified the input file several times to obtain the best results for the docking pose with the most negative binding affinity. Exhaustiveness values were set as 8. The center of the grid box was set according to the binding pocket site in the crystal structure. After the molecular docking, the ligand–receptor complexes were further analyzed using Discovery Studio Visualizer v.20 (https://www.3ds.com/, accessed on 5 September 2022).

## 4. Conclusions

One approach to the evaluation and characteristics of new therapeutic agents is an examination of binding to plasma proteins, mainly to serum albumin, which provides valuable data on the behavior of these pro-drugs in organisms. This study presented the interaction of positively charged peptides, L1–3, with major important blood plasma proteins, albumin (BSA), α1-acid glycoprotein (AAG), and γ-globulin fraction (GGF). The fluorescence spectroscopy study shows that the investigated compounds exhibit a connection with BSA and GGF except for the AAG where the fluorescence intensity nearly does not change after the ligands’ titration. The analysis of the quenching rate constant (k_q_) points obtained values that were much greater than the maximum scatters collision; therefore, the interactions of BSA and GGF with ligands L1–3 were carried out by a static mechanism and the analyzed proteins formed complexes with the studied peptides. The comparison of the binding constants showed that K_b_ is the greatest for the GGF/L1 complex (10.1 × 10^4^ dm^3^mol^−1^), and in the case of the BSA complexes, the highest values were determined after L2 compound interaction (3.39 × 10^4^ dm^3^mol^−1^). The determination of the thermodynamic negative parameters, the enthalpy (ΔH°), and entropic (ΔS°) changes, affirmed the complexation process via hydrogen bonding and/or van der Waals forces. These interactions between both BSA and GGF with L1–3 are spontaneous processes in view of obtaining negative values for the free energy change (ΔG°). The secondary structure of BSA after interaction with the studied peptides was not damaged too much. The spectroscopic methods showed destabilization on the α-helix structure in BSA after interaction with studied ligands, and the compound L1 exhibited the greatest effect. This change was contributed to by an increase in the β structures and disordered random forms. The compounds L1–3 resulted in a reduction in the proportion of the β-sheet and α-helix structure with a simultaneous increase in the random coil structure after interaction with GGF. The complex with L1 apparently showed this. The molecular docking studies indicated that three binding sites of BSA are capable of anchoring L1–3. The binding affinity to BSA for L1–3 molecules showed site 1 in Domain I is the most preferred cavity, but due to the slight difference in energy, site 2 in Domain III is also a possible place. The positively charged surfaces of L1–3, and the special contact with the side chain of Dab, Orn, and Lys, are responsible for salt bridge contacts with proteins. Moreover, much hydrogen bonding took place in complexation.

In summation, all these findings show that the L1 ligand has the strongest connection with γ-globulin fraction (GGF). The eminently highest values of the binding constant (K_b_), and the change in the entropic (ΔS°) reaction, which is a measure of disorder, were obtained for the GGF/L1 layout. It is in good agreement with the analysis of changes in the secondary structure, where the GGF/L1 complex exhibited 22.98% differences. The same influence rules were observed in the case of the L1 interaction with BSA.

## Figures and Tables

**Table 1:** The Stern–Volmer constant, K_sv_, quenching rate constant, k_q_, binding constant, K_b_, number of binding sites, n, and thermodynamic parameters for the interaction of BSA with the studied compounds at different temperatures.

		Quenching		Binding			Thermodynamic		
	T (K)	K_sv_ × 10^5^ (dm^3^mol^−1^)	k_q_ × 10^13^ (dm^3^mol^−1^s^−1^)	logK_b_	K_b_ × 10^4^ (dm^3^mol^−1^)	n	ΔG° (kJmol^−1^)	ΔH° (kJmol^−1^)	ΔS° (Jmol^−1^K^−1^)
**L1**	297 303 308	1.55 ± 0.07 1.21 ± 0.03 0.70 ± 0.06	1.55 ± 0.07 1.21 ± 0.03 0.70 ± 0.06	4.07 ± 0.07 3.95 ± 0.09 3.72 ± 0.15	1.17 ± 0.02 0.89 ± 0.02 0.53 ± 0.02	0.80 ± 0.02 0.80 ± 0.04 0.80 ± 0.02	−23 ± 1	−54 ± 2	−106 ± 3
**L2**	297 303 308	1.41 ± 0.02 1.39 ± 0.06 1.12 ± 0.07	1.41 ± 0.02 1.39 ± 0.06 1.12 ± 0.07	4.53 ± 0.08 4.04 ± 0.17 3.74 ± 0.15	3.39 ± 0.06 1.10 ± 0.05 0.55 ± 0.02	0.89 ± 0.02 0.81 ± 0.04 0.77 ± 0.04	−25 ± 1	−126 ± 1	−339 ± 3
**L3**	297 303 308	1.53 ± 0.03 1.22 ± 0.05 1.13 ± 0.06	1.53 ± 0.03 1.22 ± 0.05 1.13 ± 0.06	4.40 ± 0.12 4.10 ± 0.15 3.88 ± 0.16	2.52 ± 0.07 1.13 ± 0.04 0.76 ± 0.03	0.86 ± 0.02 0.83 ± 0.04 0.80 ± 0.03	−25 ± 1	−83 ± 1	−195 ± 2

**Table 2:** The Stern–Volmer constant, K_sv_, quenching rate constant, k_q_, binding constant, K_b_, number of binding sites, n, and thermodynamic parameters for the interaction of GGF with the studied compounds at different temperatures.

		Quenching		Binding			Thermodynamic		
	T (K)	K_sv_ × 10^4^ (dm^3^mol^−1^)	k_q_ × 10^12^ (dm^3^mol^−1^s^−1^)	logK_b_	K_b_ × 10^4^ (dm^3^mol^−1^)	n	ΔG° (kJmol^−1^)	ΔH° (kJmol^−1^)	ΔS° (Jmol^−1^K^−1^)
**L1**	297 303 308	4.24 ± 0.06 4.03 ± 0.13 3.60 ± 0.06	4.24 ± 0.06 4.03 ± 0.13 3.60 ± 0.06	5.00 ± 0.12 4.79 ± 0.20 4.39 ± 0.21	10.10 ± 0.24 6.17 ± 0.25 2.51 ± 0.12	1.06 ± 0.02 1.03 ± 0.06 0.97 ± 0.04	−29 ± 1	−94 ± 2	−220 ± 7
**L2**	297 303 308	3.99 ± 0.10 2.11 ± 0.12 1.00 ± 0.10	3.99 ± 0.06 2.11 ± 0.12 1.00 ± 0.10	4.12 ± 0.17 3.50 ± 0.20 3.31 ± 0.30	1.32 ± 0.15 0.32 ± 0.02 0.21 ± 0.02	0.89 ± 0.02 0.85 ± 0.05 0.88 ± 0.05	−23 ± 1	−130 ± 4	−363 ± 11
**L3**	297 303 308	3.87 ± 0.10 3.12 ± 0.14 2.65 ± 0.11	3.87 ± 0.10 3.12 ± 0.14 2.65 ± 0.11	4.75 ± 0.17 4.45 ± 0.19 3.83 ± 0.30	5.63 ± 0.20 2.82 ± 0.08 0.68 ± 0.01	0.86 ± 0.02 0.83 ± 0.04 0.80 ± 0.03	−27 ± 1	−144 ± 6	−393 ± 15

**Table 3:** The binding constant of L1–3/BSA system in the presence of site markers phenylbutazone (PHB) and ibuprofen (IBP) at 297 K.

Site Marker	logK_b_		
	L1	L2	L3
-	4.07 ± 0.07	4.53 ± 0.08	4.40 ± 0.12
BSA + PHB (site I)	4.00 ± 0.12	4.30 ± 0.20	4.30 ± 0.26
BSA + IBP (site II)	4.03 ± 0.15	4.42 ± 0.30	4.07 ± 0.31

**Table 4:** The percentage of content of secondary structure elements in BSA in the absence and presence of peptides L1, L2, and L3, calculated in the CD Multivariate SSE program.

BSA: Analyzed. Peptide Molar Ratio	% α-Helix	% β-Sheet	% β-Turn	% Other
**L1**				
**1:0**	60.7%	5.2%	9.9%	24.1%
**1:0.25**	59.4%	6.0%	10.0%	24.6%
**1:0.5**	59.2%	5.9%	10.1%	24.8%
**1:0.75**	58.6%	6.3%	10.1%	25.0%
**1:1**	58.5%	6.4%	10.1%	25.0%
**1:1.5**	58.2%	6.6%	10.1%	25.1%
**L2**				
**1:0**	61.9%	4.7%	9.9%	23.5%
**1:0.25**	61.2%	5.1%	9.9%	23.7%
**1:0.5**	60.9%	5.5%	9.9%	23.7%
**1:0.75**	60.9%	5.5%	9.9%	23.6%
**1:1**	60.7%	5.8%	9.9%	23.6%
**1:1.5**	60.7%	5.9%	9.9%	23.5%
**L3**				
**1:0**	61.3%	4.9%	9.9%	23.9%
**1:0.25**	60.9%	5.3%	10.0%	23.8%
**1:0.5**	60.9%	5.4%	10.0%	23.8%
**1:0.75**	61.1%	5.1%	9.9%	23.9%
**1:1**	61.0%	5.3%	9.9%	23.9%
**1:1.5**	60.9%	5.2%	9.9%	23.9%

**Table 5:** The percentage of content of secondary structure elements in GGF in the absence and presence of peptides L1, L2, and L3, calculated in the CD Multivariate SSE program.

GGF: Analyzed Peptide Molar Ratio	% α-Helix	% β-Sheet	% β-Turn	% Other
**L1**				
**1:0**	8.3%	37.9%	13.2%	40.6%
**1:0.25**	7.9%	38.0%	13.2%	40.9%
**1:0.5**	7.9%	38.0%	13.2%	40.9%
**1:0.75**	7.6%	37.8%	13.3%	41.3%
**1:1**	7.4%	37.9%	13.3%	41.4%
**1:1.5**	7.5%	37.7%	13.3%	41.5%
**L2**				
**1:0**	8.4%	37.6%	13.2%	40.7%
**1:0.25**	8.3%	37.7%	13.2%	40.8%
**1:0.5**	8.3%	37.6%	13.2%	40.8%
**1:0.75**	8.3%	37.7%	13.2%	40.7%
**1:1**	8.1%	37.9%	13.2%	40.8%
**1:1.5**	7.5%	37.8%	13.3%	41.4%
**L3**				
**1:0**	8.2%	37.6%	13.2%	41.0%
**1:0.25**	8.0%	37.7%	13.2%	41.1%
**1:0.5**	7.8%	38.0%	13.2%	40.9%
**1:0.75**	7.8%	38.0%	13.2%	41.0%
**1:1**	7.4%	37.9%	13.3%	41.5%
**1:1.5**	7.0%	38.0%	13.3%	41.7%

**Table 6:** The percentage of the secondary structure contribution of free BSA, GGF, and their mixture with L1, L2, L3 obtained by I Amide band deconvolution.

Protein/Complex	(%)				
	α-Helix	β-Sheet	β-Turn	β-Anti	Random Coil
free BSA	63.71	1.64	6.65	2.38	25.62
BSA/L1					
1:0.5	61.36	3.87	7.47	0.77	26.54
1:1.0	49.45	8.28	13.86	0.49	27.93
1:1.5	49.40	8.69	13.99	0.31	27.61
BSA/L2					
1:0.5	55.96	3.31	8.42	2.54	29.77
1:1.0	55.19	3.17	9.59	2.56	29.49
1:1.5	54.12	3.00	10.15	2.81	29.93
BSA/L3					
1:0.5	55.00	3.31	9.86	2.35	29.47
1:1.0	55.31	3.19	9.40	2.68	29.43
1:1.5	54.40	3.52	9.71	2.69	29.69
free GGF	14.17	47.7	14.55	5.73	17.69
GGF/L1					
1:0.5	11.67	34.54	10.12	3.85	39.82
1:1.0	9.41	35.36	11.38	4.13	39.71
1:1.5	9.37	35.72	10.12	4.12	40.67
GGF/L2					
1:0.5	10.54	43.42	8.55	3.22	34.28
1:1.0	9.59	44.29	8.48	4.31	33.33
1:1.5	9.54	43.35	8.35	4.86	33.91
GGF/L3					
1:0.5	13.84	40.58	17.16	6.23	22.19
1:1.0	12.52	40.11	18.63	5.53	23.22
1:1.5	12.08	40.70	18.16	5.01	24.05

**Table 7:** The binding affinity (kcal/mol) for interaction L1–3 with serum albumin and γ-globulin fraction.

	Serum Albumin			γ-Globulin Fraction
	Domain I (Site 3)	Domain II (Site 1)	Domain III (Site 2)	
**L1**	−5.5	−4.6	−5.3	−5.5
**L2**	−6.1	−4.4	−5.9	−6.1
**L3**	−6.2	−4.2	−5.8	−5.4

## Data Availability

Not applicable.

## Abbreviations

AAG, α1-acid glycoprotein; ATR-FTIR, attenuated total reflectance Fourier-transform infrared spectroscopy; BSA, bovine serum albumin; CAP, cationic antimicrobial peptides; CD, circular dichroism; DTGS, deuterated triglicyne sulphate; GGF, γ-globulin fraction; IBP, ibuprofen; IR, infrared spectroscopy; NRP, non-ribosomal peptides; PHB, phenylbutazone; ROS, reactive oxygen species.
